# Predicting *EGFR* mutational status from pathology images using a real-world dataset

**DOI:** 10.1038/s41598-023-31284-6

**Published:** 2023-03-16

**Authors:** James J. Pao, Mikayla Biggs, Daniel Duncan, Douglas I. Lin, Richard Davis, Richard S. P. Huang, Donna Ferguson, Tyler Janovitz, Matthew C. Hiemenz, Nathanial R. Eddy, Erik Lehnert, Moran N. Cabili, Garrett M. Frampton, Priti S. Hegde, Lee A. Albacker

**Affiliations:** Foundation Medicine Inc., 150 Second Street, Cambridge, MA USA

**Keywords:** Machine learning, Image processing

## Abstract

Treatment of non-small cell lung cancer is increasingly biomarker driven with multiple genomic alterations, including those in the epidermal growth factor receptor (*EGFR*) gene, that benefit from targeted therapies. We developed a set of algorithms to assess *EGFR* status and morphology using a real-world advanced lung adenocarcinoma cohort of 2099 patients with hematoxylin and eosin (H&E) images exhibiting high morphological diversity and low tumor content relative to public datasets. The best performing *EGFR* algorithm was attention-based and achieved an area under the curve (AUC) of 0.870, a negative predictive value (NPV) of 0.954 and a positive predictive value (PPV) of 0.410 in a validation cohort reflecting the 15% prevalence of *EGFR* mutations in lung adenocarcinoma. The attention model outperformed a heuristic-based model focused exclusively on tumor regions, and we show that although the attention model also extracts signal primarily from tumor morphology, it extracts additional signal from non-tumor tissue regions. Further analysis of high-attention regions by pathologists showed associations of predicted *EGFR* negativity with solid growth patterns and higher peritumoral immune presence. This algorithm highlights the potential of deep learning tools to provide instantaneous rule-out screening for biomarker alterations and may help prioritize the use of scarce tissue for biomarker testing.

## Introduction

Genomic-guided therapeutic choices are increasingly used in the management of advanced non-small cell lung cancer (NSCLC)^1^. Therapies requiring diagnostic testing include single-agent immunotherapy and kinase inhibitors targeting EGFR and ALK in the first-line and KRAS G12C, MET, and NTRK targeted therapies in the second-line^2^. Although multiplex diagnostic approaches such as next-generation sequencing are becoming more common, many labs perform testing for relevant biomarkers separately. As tissue acquired for testing is often limited and the number of diagnostics increases, care should be taken to prevent tissue exhaustion so that all appropriate clinical options may be determined^3^. One potential opportunity to mitigate this challenge is by leveraging machine learning with digital pathology.

Machine learning, and in particular deep learning, has recently gained broad traction across an expanse of medical domains, with its use showing promise in aiding diagnostics and biomarker discovery in applications relating to ophthalmology, heart disease, cancer care and more^4–11^. There is especially impactful opportunity within cancer care to leverage the immense data generated through clinical practice, including omics from sequencing technologies and gigapixel digital pathology scans. One such opportunity lays with the emerging sub-field of digital pathology, which investigates the rich trove of information present within high resolution scans of hematoxylin and eosin (H&E) stains alongside other stains such as immunohistochemistry stains. H&E stains are inexpensive and ubiquitous tissue specimen stains used during the pathology workflow that allow pathologists to better examine tumor morphologies and determine the diagnosis of the tumor^12^. Machine learning and deep learning models applied to digital scans of H&E-stained tissue slides have shown significant promise in enhancing a variety of aspects in cancer-care, including aiding in cancer diagnoses, improving operational efficiencies, and directly providing molecular insights.

In 2016, Wang et al. showed that deep learning could detect metastatic breast cancer in lymph node biopsies with high performance, and suggested value in computer-aided approaches augmenting the pathology workflow, with pathologist-computer combined methods achieving 0.995 AUC on the cancer detection task^13^. Following soon afterwards, Coudray et al. showed that deep learning could classify cancer subtypes effectively and, even more promisingly, could also predict gene alterations directly from lung adenocarcinoma H&E images, achieving 0.733 to 0.856 AUC for mutations in *STK11*, *EGFR*, *FAT1*, *SETBP1*, *KRAS* and *TP53*^14^. In 2018, Ilse et al. proposed that histopathology problems could be effectively formulated as multiple-instance problems, which many studies have applied to address a range of histopathology problems^15^. Campanella et al. showed that multiple-instance approaches could achieve clinical-level performance on predicting prostate and other cancers and, from a biomarker perspective, Naik et al. showed that such approaches could predict estrogen receptor status from breast cancers with high performance (0.92 AUC)^16,17^.

Despite these advances, clinical adoption of machine learning in digital pathology has been slow. This may be partially due to a lack of clinically relevant datasets for research, with many research models trained on homogenous datasets with high tumor purity^14,18^. For example, The Cancer Genome Atlas’s requirement of 60% tumor purity in most diseases is in stark contrast to more real-world settings where tumor purities of 20% are common^19^. Further limiting clinical use is the challenge of interpreting predictions made by deep learning models, making it difficult to ensure that given models are relevant and accurate for specific clinical samples.

Here we demonstrate that attention-based multiple-instance learning can predict *EGFR* mutational status in advanced metastatic lung adenocarcinoma samples directly from H&E images with state-of-the-art performance on real-world datasets, where many samples have less than 50% tumor content. Through a combination of tissue morphology classification models and pathologist review we show that although tumor regions contain the most signal for *EGFR*, the attention-based model also considers relevant outlier instances from other tissue types such as immune or stromal features when predicting *EGFR* mutational status. With additional analysis via association rules mining, we demonstrate a process wherein morphology models and pathologist expertise can be leveraged to biologically verify end-to-end biomarker predictions by evaluating associated feature combinations, allowing for better model interpretation when supporting clinical decisions.

## Results

**Figure 1:**
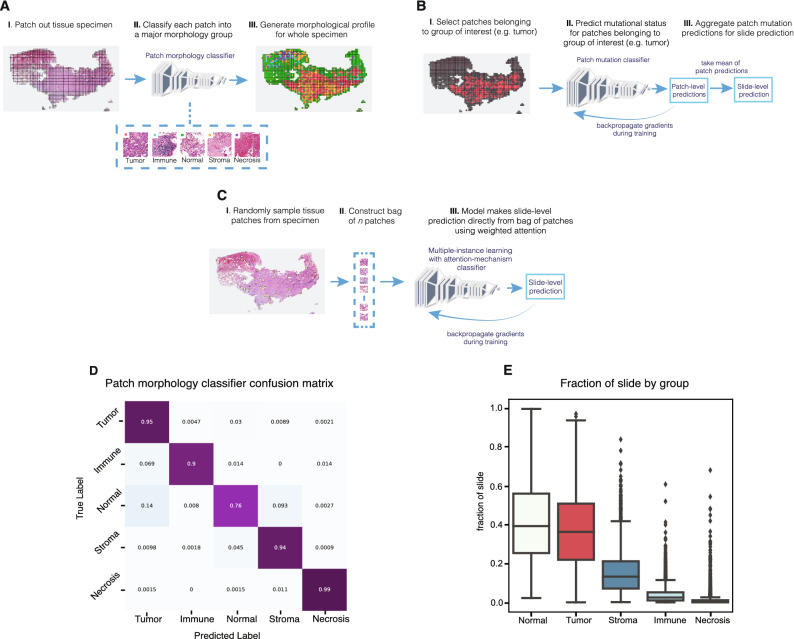
Various models to characterize and predict *EGFR* mutational status from specimens with highly diverse tissue morphologies. **(a)** A deep learning tissue morphology model that produces broad patch-level classifications for all patches from a slide. **(b)** A deep learning modeling approach to predict *EGFR* mutational status at a slide-level by utilizing patches belonging to a specific predicted tissue group only and aggregating patch predictions for the final output. **(c)** A multiple-instance learning approach that utilizes an attention mechanism, allowing for machine-intuition to automatically weigh patches of interest when directly predicting *EGFR* mutational status for the slide using a bag of patches. **(d)** Confusion matrix for the patch-level tissue morphology classifier that categorizes each patch given its predominant tissue morphology. **(e)** The distribution of tissue morphologies exhibited across the real-world lung adenocarcinoma cohort (n = 2099) as classified by the tissue morphology model.

We investigated the ability of different modeling approaches to predict *EGFR* mutational status in lung adenocarcinoma resections (see “Methods” for details). First, to set a modeling baseline, we trained a weakly-supervised model that predicts *EGFR* mutational status using all tissue patches from a slide, irrespective of morphology, and aggregates those patch predictions to generate a slide-level output. Second, we trained a two-stage model. Stage one was a convolutional neural network, which classified patch morphology into categories of tumor, immune foci, stroma, necrosis, or normal tissue (Fig. 1a). Stage two models use only tissue patches classified from a single group (Fig. 1b). Last, we trained a multiple-instance learning model that achieves state-of-the-art performance for predicting *EGFR* mutational status in lung adenocarcinoma resections and investigated the features learned by the model (Fig. 1c).

### Real-world lung adenocarcinoma samples exhibit high morphological diversity

Our experimental dataset was comprised of 2099 lung adenocarcinoma resections from advanced or metastatic cancer patients whose specimens were submitted to Foundation Medicine for genomic profiling. Of the 2099 resections, 716 (34%) were *EGFR* mutated (see “Methods”). The remainder of the dataset consisted of non-*EGFR* driver mutated specimens (e.g. *KRAS* or *ALK*) or driver wild-type specimens.

To evaluate the extent of morphological diversity within the samples comprising our dataset, we used pathologist annotations of tissue types to train a deep learning model that classifies tissue patches (512 × 512 pixels at 20 × magnification, resized to 448 × 448 pixels) into one of five tissue morphology groups: tumor, immune foci, stroma, necrosis, and normal. These predictions capture the predominant tissue type within a patch, but multiple cell types are likely present. The model effectively discriminated tumor patches from other tissue types, achieving a validation f1-score of 0.961 (4504/4658). Performance across all groups was high as shown by the f1-scores for stroma (0.943; 2106/2233), immune (0.897; 262/292), and necrotic tissue (0.986; 1276/1294) (Fig. 1d). Normal tissue had the lowest f1-score of 0.727 (570/784) with 0.093 (35/376) of normal patches predicted as stroma and 0.138 (52/376) predicted as tumor.

By applying this tissue morphology classification model to all tissue slides, we determined that most slides have a high fractional area of non-tumor tissue types (Fig. 1e). Across the dataset, the median tumor fraction by patch area was 0.364 with an interquartile range of 0.290. Notably, the patch area of normal tissue had a median fraction of 0.394 with an interquartile range of 0.307, higher than that of the tumor group. There was an appreciable presence of stroma, with a median fraction of 0.133 and an interquartile range of 0.141. Immune and necrosis patches were present with median fractions of 0.025 and 0.002 and interquartile ranges of 0.042 and 0.011, respectively. Thus, relative to many research datasets like The Cancer Genome Atlas, our tissue slides exhibit high morphological diversity and low tumor content.

### Human-intuition models help isolate predictive signal when modeling morphologically-diverse real-world data

To provide a baseline model for predicting *EGFR* mutational status from our dataset, we trained a weakly-supervised patch-level classification model using ResNet50 as a backbone with five-fold cross-validation. The weakly-supervised models obtained an AUC of 0.792 ± 0.029 when aggregating patch predictions by the slide average and an AUC of 0.784 ± 0.026 when aggregating using the median.

Since *EGFR* is a tumor cell-intrinsic driver alteration, we hypothesized that tumor regions would contain most of the classification signal. We tested this hypothesis by developing a two-stage approach, first separating patches into the five tissue morphology types and then training separate deep learning classifiers to predict *EGFR* status using only patches from one of the morphology types. Each of these five morphology-selective models were trained with five-fold cross-validation.

**Figure 2:**
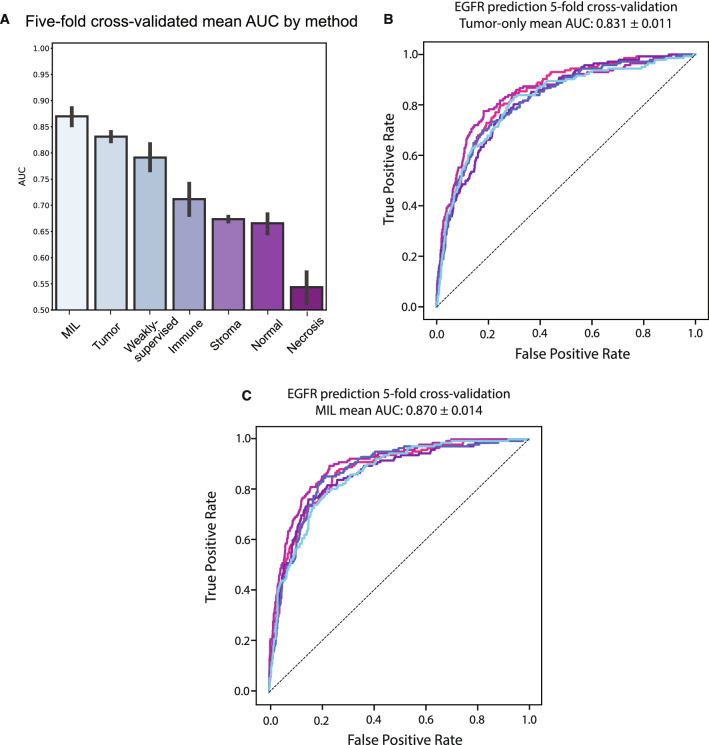
Performance for deep learning models to predict tissue morphology and *EGFR* mutational status. **(a)** Comparison of all deep learning models for predicting *EGFR* status from H&E images. **(b,c)** Cross-validated receiver operator characteristic curve for each cross-fold of **(b)** the tumor-only two-stage *EGFR* model and **(c)** the MIL model.

The tumor patch-based models achieved an AUC of 0.831 ± 0.011 when aggregating using average patch prediction and an AUC of 0.828 ± 0.009 when aggregating using median patch prediction, which was better than the weakly-supervised models (Fig. 2a,b; *p* = 0.033).

In comparison, the cross-validated AUCs by mean aggregation for the immune (0.712 ± 0.039), stroma (0.673 ± 0.005), normal (0.666 ± 0.022), and necrosis (0.544 ± 0.034) based models were significantly worse than the tumor-based models and the weakly-supervised models (Fig. 2a; *p* = 0.012, *p* = 4.62e−5, *p* = 1.30e−4, *p* = 4.083e−6). We conclude that when training patch-based models, tumor regions contain the highest signal for *EGFR* classification and that excluding non-tumor regions from consideration reduces noise and increases performance.

### Multiple-instance learning model using attention mechanism improves *EGFR* predictive ability

We next assessed an attention-based multiple-instance learning (MIL) model to determine whether *EGFR* prediction performance could be further improved using machine-intuition alone. Bags of patches were randomly sampled from each slide during training and the entire bag was given the specimen-level *EGFR* status as the label. Through the attention mechanism, the model learned without human guidance how to weigh different patches within each bag when predicting for specimen-level mutational status. The AUC achieved by the MIL models with five-fold cross-validation was 0.870 ± 0.014, which was significantly higher than the tumor-only models (Fig. 2a,c; *p* = 0.002). The models also achieved an NPV of 0.954 ± 0.024 and a PPV of 0.41 ± 0.081 at a binary classification threshold of 0.5. If only slides with high-confidence predictions (defined as < 0.25 for wild-type call and > 0.75 for mutant call) were considered, the NPV was 0.970 ± 0.017 and the PPV was 0.527 ± 0.088. Thus, attention-based models outperformed the human-guided tumor-only models.

**Figure 3:**
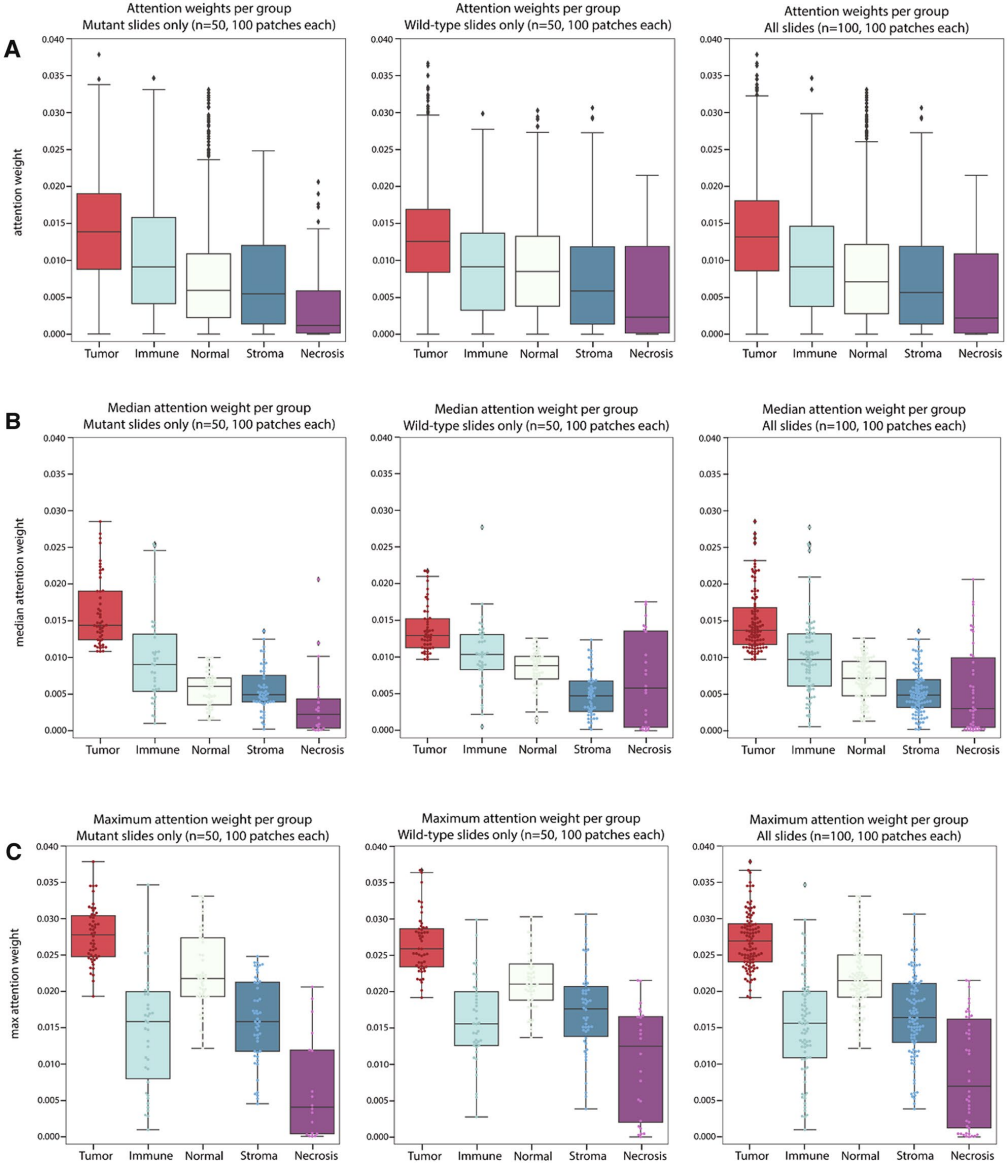

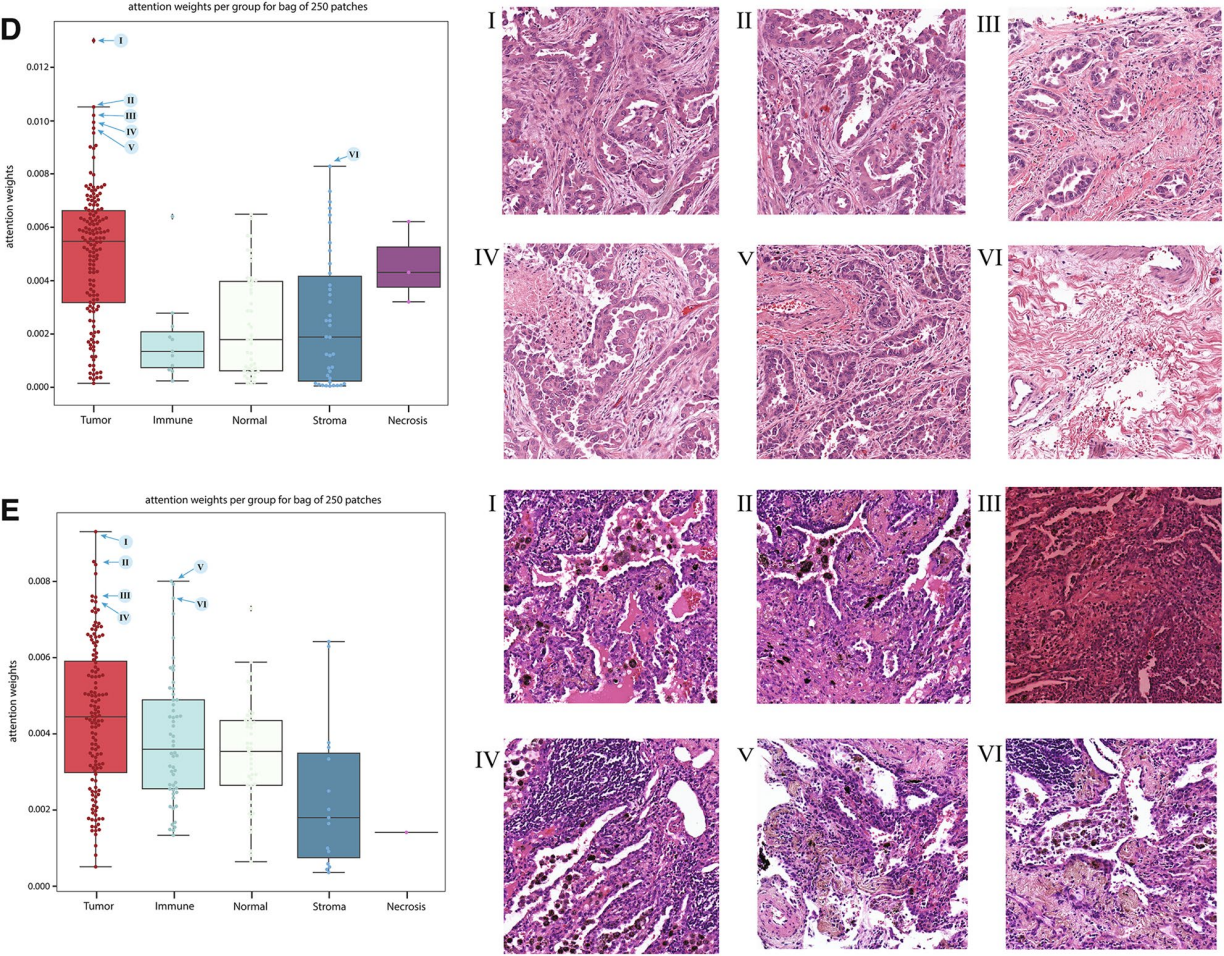
Bags from slides with high-confidence predictions assessed by the MIL model, with attention weights extracted for each patch within the bags. **(a)** All patch**, (b)** median per slide, and **(c)** maximum per slide attention weights for *EGFR* prediction as separated by predicted tissue morphology from (left column) 50 *EGFR* mutant slides, (center column) 50 *EGFR* wild-type slides, and (right column) 100 slides combined. **(d)** *EGFR* TP exemplar with attention weights from bag of 250 patches. The six highest attention patches are shown (I-VI). **(e)** *EGFR* TN exemplar with attention weights from bag of 250 patches. The six highest attention patches are shown (I-VI).

We next investigated regions with high attention scores from the MIL models to better understand what features the MIL models learned. We sampled 100 patches per bag for 100 validation slides and assessed model attention by tissue morphology (Fig. 3a–c). We found that the median attention score was highest for tumor patches at 0.013 with a maximum score of 0.038. Tumor patches received the highest attention when assessing all patches, median per slide, or maximum per slide attention. As a group, the immune patches were second with a median attention score of 0.009 and a maximum of 0.035. The median attention given to normal patches, stroma patches, and necrosis patches were 0.007, 0.006, and 0.002 with corresponding maximum attention scores of 0.033, 0.031, and 0.022, respectively. The tissue morphology classification of patches also allowed pathologists to quickly assess high-attention outlier patches for noteworthy visual features (Fig. 3d,e). In Fig. 3d, an *EGFR* true positive exemplar is presented. High attention was given to tumor and stroma patches. Patches I-V had a predominant acinar pattern and hobnail cytology, with low peritumoral and intratumoral immune fractions, ranging from 0.1 to 0.2. Patch IV had a low presence of necrotic tissue and patch VI was predicted as stroma by the tissue-morphology model, and pathologists confirmed this patch was fibrosis. In Fig. 3e, an *EGFR* true negative exemplar is presented. High attention was given to tumor patches and some immune patches. Patches I–II showed an acinar/lepidic pattern with hobnail cytology and intratumoral lymphoid aggregates. Patches III–VI were predicted to be tumor or immune foci by the tissue-morphology model. Pathologists confirmed high peritumoral and intratumoral immune fraction, ranging from 0.2 to 0.7, for these patches. Inflammation was noticeably present as well in patch IV. From these data we conclude that the MIL models learned to give high attention to tumor regions but likely boosted performance by also giving high attention to additional patterns that aid in classification such as immune infiltrates in *EGFR* negative samples.

**Figure 4:**
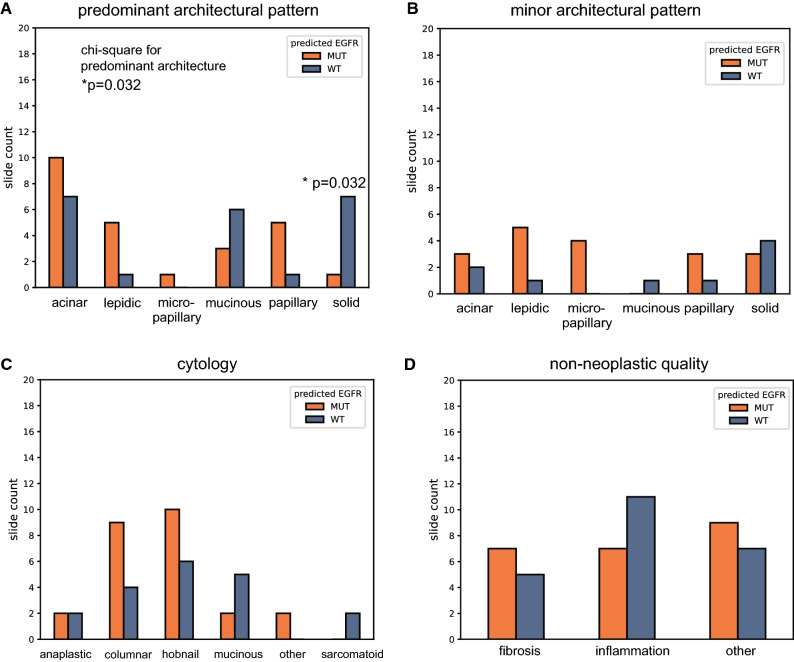
Overall bag characteristics of high-attention patches for categorical variables for 49 pathologist reviewed bags. **(a)** Predominant architectural pattern of high-attention patches, determined by patch mode, by predicted status. *p*-value from a Chi-squared test of the overall distribution. **(b)** Minor architectural pattern of high-attention patches. **(c)** Cytology for high-attention patches, determined by patch mode. **(d)** Non-neoplastic qualities present in high-attention patches, as determined by patch mode.

Pathologists also reviewed the top-25 highest attention patches in each of 49 randomly sampled bags for which the MIL models produced high confidence predictions. Bags predicted to be *EGFR* mutant had a lower standard deviation of tumor nuclei fraction across the highest-attention patches (Supplementary Table 1; *p* = 0.028, Pearson’s r: − 0.317). Bags predicted to be *EGFR* mutant also had higher minimum tumor nuclei fraction (*p* = 0.037, Pearson’s r: 0.301) and lower maximum peritumoral immune fraction (*p* = 0.041, Pearson’s r: − 0.297). Pathologists also assigned tumor architectural patterns to high attention patches. The overall mode of predominant tumor architectural patterns exhibited a statistically significant difference between *EGFR* mutant and wild-type slides (Fig. 4a; *p* = 0.035; Chi-squared test). More bags were predicted to be wild-type than *EGFR* mutant when the predominant architectural pattern was solid (Fig. 4a; *p* = 0.013).

There were also several trends in the data that are suggestive of known associations with *EGFR* mutational status that did not reach statistical significance. When considering overall architecture, bags that were predominantly lepidic or papillary were predicted as *EGFR* mutant five times more often than *EGFR* wild-type (Fig. 4a). In contrast, bags that predominantly possessed the solid architecture were predicted as *EGFR* wild-type seven times more often than mutant. When the predominant architecture was mucinous, it was twice as likely that the bag would be predicted as *EGFR* wild-type. There was no strong enrichment (ratio < 2.0) in prediction status of either type for predominantly acinar bags. All bags with any micropapillary content (predominant or minor) were predicted as *EGFR* mutant specimens (Fig. 4a,b). The directionality of preference for predicted status when considering acinar, lepidic, papillary, mucinous for the minor architectures were similar to the preference in the predominant architecture, but the solid minor architectural pattern did not see the same strength of preference for *EGFR* mutant predictions compared to instances where the solid architecture was the predominant pattern. From a cytology perspective, bags with columnar or hobnail as the most common cell type across the high-attention patches were more likely (> 1.5) to be predicted as mutant (Fig. 4c). Mucinous and sarcomatoid cytologies were more likely to be predicted as wild-type. From an overall tumor-feature perspective, our MIL models tended to predict lepidic and papillary patterns as *EGFR* mutant and any mucinous characteristic (architecture and cytology) as *EGFR* wild-type (Fig. 4c). For non-neoplastic qualities, slides with inflammation were more frequently predicted as *EGFR* wild-type (Fig. 4d). Generally, there were no categorical characteristics (aside from the micropapillary pattern) that perfectly separated specimens by predicted status, possibly suggesting that the models consider the various characteristics within each bag in combination.

To examine the relevance of the patch characterization in a combinatorial manner, we performed association rules mining^21^ to determine item-sets of interest using the categorical variables (Supplementary Table 2). Each bag’s overall characterization was determined via the category mode for the reviewed patches in the bag. The highest-lift item-sets for predicted wild-type status as a consequent included: {inflammation, hobnail cytology, solid minor architectural pattern}, {inflammation, acinar predominant architectural pattern, hobnail cytology}, {acinar predominant architectural pattern, hobnail cytology, solid minor architectural pattern} and {acinar predominant architectural pattern, inflammation, hobnail cytology, solid minor architectural pattern}, each with a lift of 2.097. In contrast, the highest-lift item-sets for predicted *EGFR* mutated status included: {fibrosis, lepidic minor architectural pattern, hobnail cytology} and {fibrosis, acinar predominant architectural pattern, hobnail cytology}, both with a lift of 1.92. In total, the *EGFR* prediction algorithm recapitulated several known morphological and cytological associations with *EGFR* status and these features can be tested on a per sample basis by analyzing highly attended regions manually or via tissue morphology/cytology classification algorithms.

## Discussion

As barriers to clinical adoption of digital tools are reduced, the development of machine learning models to augment and support established processes is highly desirable. However, models trained on research datasets that are dissimilar to real-world data may have difficulty generalizing in a clinical setting, where the incoming sample distribution may not align well with the training data. With this in mind, we developed machine learning models that predict *EGFR* mutational status on real-world H&E lung adenocarcinoma images with high morphological diversity and show the potential for use as screening algorithms with high NPV. We demonstrate that state-of-the-art performance for predicting *EGFR* can be achieved by using attention-based models that evaluate a full range of tissue morphologies, outperforming our tumor-only models as well as those shown in prior literature (0.825–0.831 AUC)^14^. Additionally, attention-based models do not require expensive manual annotation or guidance to train. Finally, we show that biological verification of attention-based end-to-end models can be performed by combining assessment approaches such as morphological profiling, item-set analysis, and pathology review, potentially increasing accuracy in a clinical setting.

The ability to directly assess the attention distribution of MIL models also allows an opportunity to investigate learned patterns regarding tumor biology and the tumor microenvironment (TME) when predicting *EGFR* mutational status. Various studies have shown that certain tumor architectural patterns and cytological features are correlated with *EGFR* mutated tumors in lung adenocarcinoma. For example, Sun et al. showed that acinar and lepidic architectural patterns, sometimes in mixed combination, are associated with *EGFR* mutations in NSCLC^22^. Other studies show that micropapillary or papillary patterns, with any presence of the lepidic pattern, are good indicators of *EGFR* mutation^23,24^. Associations of hobnail cytology with *EGFR* mutated samples have also been observed^25^. In line with these findings, our MIL models differentiate mutational status using the predominant architectural pattern (Fig. 4a), and appear to capture a relationship between *EGFR* mutations and the lepidic pattern coupled with hobnail cytology (Supplementary Table 2). It should be mentioned that the hobnail cytology is also present within high-lift wild-type prediction sets; however, the lepidic pattern is present only within high-lift predicted mutant sets while the solid and mucinous patterns are present only in the high-lift predicted wild-type sets. It is noteworthy that our models do not learn a sole architectural pattern, cytology, or non-neoplastic quality as the lone discriminator for predicting *EGFR* status.

The attention distribution of MIL algorithms also has the potential to allow for quality control tests in a clinical setting. One quality control method would be to simply have pathologists review highly attended patches and ensure they had characteristics of the *EGFR* mutant or wild-type call. A more automated approach would be to use trained morphology, growth pattern, and cytology algorithms to analyze highly attended patches. For example, an *EGFR* mutated prediction in a sample with predominantly solid architecture could be flagged and reviewed manually. If a pathologist then confirms that an *EGFR* mutation is unlikely given the specimen morphology, the specimen can be prioritized for genomic testing. Furthermore, utilizing multiple observations (or algorithms) to assess whether a particular diagnostic result is consistent with all the available evidence is similar to how pathologists assess cases in practice.

Beyond tumor-associated features, it has also been suggested that immune response and non-neoplastic components within the TME may be relevant when examining the effect of mutations upon linked biological pathways. Dong et al. showed that *EGFR* mutated NSCLC specimens possess significantly less T cell infiltration and lower immunogenicity than wild-type specimens^26^. In another study, Lin et al. suggest that TME immune response may be influenced by the *EGFR* mutation via manipulation of complex signaling pathways, leading to a reduction in the expression of the major histocompatibility complex and consequently lowered activation levels of CD8 + T cells^27^. Our MIL models appear to learn this trend of lowered immune response within the TME of *EGFR* mutated specimens, in part indicated by the significantly higher maximum peritumoral immune fraction (Supplementary Fig. 1c; *p* = 0.041) across high-attention patches for specimens strongly predicted to be wild-type. Additionally, inflammation is present within three out of four of the highest-lift item-sets for *EGFR* wild-type predictions, while it is absent from the highest-lift item-sets for *EGFR* mutant predictions.

Finally, the ability to examine the attention given by MIL models may allow exploration of other less obvious elements within the TME that could help elucidate the biological understanding of *EGFR* mutations. In two of the highest-lift item-sets for predicted *EGFR* mutant status, fibrosis is present alongside the tumor-related features. This inclusion of fibrosis is less expected than the inclusion of tumor features but may also suggest interesting interactions within the TME. Many studies now suggest that stroma and stromal elements may play far more than a passive role within TMEs and may have direct effects on tumorigenesis. For example, cancer-associated fibroblasts within desmoplastic stroma may help promote tumor invasion and metastasis, oncogenic angiogenesis, and immune evasion^28^. One change within the TME possibly affected by activated fibroblasts is the assisted generation and structuring of the extracellular matrix, which may influence tumor growth and cell motility^29,30^. Additionally, stroma may play a role in immune evasion by acting as a physical barrier to T cell infiltration^31^. The inclusion of fibrosis as a relevant feature may indicate the ability of machine learning models to recognize, without human guidance, patterns involving tissue regions that may be orthogonal to tumor-specific features.

Our experiments show that machine learning models enabled with self-directed intuition such as attention-based MIL models can predict *EGFR* mutational status, and potentially other biomarkers, from morphologically-diverse real-world tissue specimens without human intervention. The ability to rely upon machine-intuition to extract meaningful features could enable low-effort signal-searching experiments at scale, as well as provide a means to investigate machine-discovered patterns within the phenotype that may be biologically informative. It is encouraging from an interpretability standpoint that models intended to assist in clinical decision-making recapitulate expected results, such as finding tumor regions most predictive for genomic alteration signal, but also that such models may be capable of determining patterns and interactions within phenotypic features in ways that elevate performance beyond methods relying solely upon human intuition. In a clinical setting, these screening algorithms could provide rapid genomic insights regarding a patient specimen, which can then be checked by a combination of more interpretable models as well as pathologist visual examination. Any low-confidence predictions or samples flagged by pathologists could then be selected for further genomic testing.

We do note that the samples used within this study were limited to lung adenocarcinoma resections that were extracted from lung tissue sites only. Oftentimes in clinical practice, a majority of specimens are acquired as needle core biopsies from a variety of tissues outside of and including the lungs. To increase the clinical utility of pre-screening algorithms such as those described in our study, approaches should be developed to integrate both resection samples and needle core biopsy samples to enable optimal coverage of the clinical patient population. Since needle core biopsies offer much less tissue than resections for analysis, thoughtful modeling approaches to reconcile this difference will be needed. In the future, we hope to expand model performance to cover needle core samples across a variety of tissue extraction sites and to evaluate model transferability on additional external clinical datasets.

## Conclusions

We developed a multiple-instance model to predict *EGFR* mutational status in lung adenocarcinoma samples with diverse tissue morphologies, achieving an AUC of 0.870 with an NPV of 0.954 and a PPV of 0.410. By using a combination of tissue morphology classification models and expert pathologist review of high-attention patches to assess signal distribution, we found that our model learns to consider both tumor morphology as well as non-tumor morphologies when predicting *EGFR* mutational status. Our model’s performance as evaluated on validation sets reflecting the real-world prevalence of *EGFR* mutations in lung adenocarcinoma suggests utility as a rule-out screening tool that could provide rapid genomic insights regarding a patient specimen.

## Methods

### Dataset

The dataset used in this study consists of lung adenocarcinoma resection H&E whole slide image scans acquired from specimens submitted to Foundation Medicine for genomic profiling. All data was de-identified following a de-identification protocol that was externally approved according to the Health Insurance Portability and Accountability Act Expert Determination Process. All images within this dataset were scanned at 20 × magnification. This image dataset was generated from 2099 tissue specimens from 2099 individual patients. 716 of the specimens were determined by genomic sequencing to be *EGFR* short-variant mutant specimens. Of the remaining specimens, 85 were *ALK* mutated, 93 *BRAF* mutated, 81 *ERBB2* mutated, 606 *KRAS* mutated, 76 *MET* mutated, 35 *RET* mutated, 18 *ROS1* mutated, and *389* were lung driver wild-type.

Five-fold cross-validation was performed to evaluate model performance and consistency. For ground-truth, all slides used the specimen-level mutational statuses as determined by FMI’s next-generation sequencing tests. The training/validation split for all experiments was 0.8/0.2 for *EGFR* mutant slides. The real-world prevalence of *EGFR* short variant mutations is approximately 15% in NSCLC, and thus represents a minority class for which class-imbalanced modeling was a consideration. As the data available at FMI contained a relatively large number of *EGFR* mutated lung adenocarcinoma specimens, we chose to forgo any minority class balancing techniques such as minority over-sampling or minority class weight penalization and instead chose to perform majority under-sampling, randomly selecting an equal number of slides that were not *EGFR* mutated to balance the *EGFR* mutated slides in the training set. For the validation sets, we selected enough slides that were not *EGFR* mutated so that the percentage of *EGFR* mutated slides in the validation was 15%, reflecting the real-world prevalence. By doing so, we aimed to simplify the training process while still allowing for an evaluation of the model against a validation dataset that more closely represented a real-world setting. As a result, each training set had 1146 slides and each validation set had 953 slides.

### Model architecture

The tissue morphology classifier was structured primarily using a trainable feature extractor (ResNet50^32^ without the top-layer). The feature extractor was followed by a global average pooling layer, which is then connected to a 5-dimensional fully-connected layer with softmax activation to predict the tissue type classification.

The weakly-supervised *EGFR* prediction model consisted of a trainable feature extractor, followed by a global average pooling layer, a dropout layer of 0.3, and a final 1-dimensional output layer with a sigmoid activation to predicted *EGFR* status at patch-level. The specimen-level prediction was made by aggregating patch-level predictions from the given slide. Each morphology-restricted patch-level *EGFR* classifier used the same architecture as the weakly-supervised model. The feature extractor backbone for all models was ResNet50.

The attention-based multiple-instance learning model was built using ResNet50 without the top-layer and with an added global average pooling layer to serve as a trainable feature extractor. Following the feature extractor was an attention-mechanism consisting of two fully-connected layers (512-dimensional, 256-dimensional) to reduce the embedding dimensionality. The reduced embeddings were then passed to a 256-dimensional fully-connected layer followed by another 1-dimensional fully-connected layer. The output is then transposed and all patches within a multiple-instance bag are passed through a softmax activation which fractionally weighs the attention for each patch within the bag. The reduced embeddings are then weighed using the softmax attention weights to generate the slide-level weighted embedding. A final fully-connected layer processes the slide-level weighted embedding and uses the sigmoid activation to predict the specimen-level *EGFR* status.

### Tissue morphology annotation procedure

In order to train a model to profile tissue types within the lung adenocarcinoma specimen set, pathologists performed non-exhaustive region annotations on a selection of lung adenocarcinoma slides for the tissue morphologies: tumor, normal lung tissue, stroma, immune foci, and necrosis. These annotations were performed to capture large representative regions for each of the groups, but since the tumor microenvironment is highly complex there are likely elements belonging to other groups within patches extracted from a particular morphology annotation.

The tissue morphology classifier was then trained to predict the tissue morphology for patches extracted from the region annotations. These annotations were deliberately chosen to maximize the variety within the morphological groups. For example, when annotating tumor regions, an effort was made to find and annotate the different lung adenocarcinoma histological subtype groups (lepidic, acinar, micropapillary, papillary, mucinous, and solid). Similarly, the scope for normal lung tissue annotations was also broad and included different sections of alveolar tissue and cartilage.

### Training procedure

To train the tissue morphology model, we generated a patch dataset by extracting non-overlapping patches directly from the pathologist-annotated regions-of-interest for the five chosen morphological classes (tumor, immune, normal, stroma, necrosis). The selection criteria for a morphology patch to be extracted was that each patch needed a pixel fraction of at least 0.75 to be from within an annotated region. We chose to extract these patches at 512 × 512 pixels at 20 × magnification, as this was determined via pathologist guidance to be adequate in receptive field for capturing the signal for the tissue morphology prediction task. We did not extract patches at a larger receptive field for this task in order to maximize the number of patches we could generate from the limited annotations we possessed. For training the tissue morphology model we resized the 512 × 512 patches to 448 × 448 patches before inputting into the model. We found that this resizing step allowed for larger batches during training and reduced the overall training time by 25% while maintaining a validation concordance greater than 95% between a morphology classifier using 448 × 448 inputs and a classifier using 512 × 512 inputs.

The patch morphology classification task was structured as a multi-class classification task. For simplicity, assume a single input instead of a batch. The morphology patch classifier takes a tissue patch as its input and classifies it into one of *K* categories. The final output layer for the morphology classifier is a fully connected layer of *K* dimensions and the activation function is the softmax function:

$$a\left( z \right)_{i} = \frac{{e^{{z_{i} }} }}{{\mathop \sum \nolimits_{j = 1}^{K} e^{{z_{j} }} }}$$

where

$$z = w^{T} x + b$$

and where *x* is the input to the fully-connected output layer, *w* is the weight matrix of the output layer, and *b* is the bias term for the output layer. The loss that we optimize for is the categorical cross-entropy loss:

$$l = - \mathop \sum \limits_{j = 1}^{K} \hat{y}_{j} log\left( {a\left( z \right)_{j} } \right)$$

where $\hat{y}_{j}$ is the target value of the *j*-th class for the sample. The morphology model was trained for 15 epochs and the model weights corresponding to the highest validation accuracy were used to morphologically profile the full resection set (n = 2099) at a patch level.

To train the *EGFR* mutation prediction models, we generated a patch dataset by exhaustively extracting non-overlapping tissue patches from all resection slides. We first performed tissue masking on down-sampled images for every slide in the resection cohort. The masking was performed on down-sampled images in the interest of computational efficiency. The tissue masking approach consisted of a colorspace transformation of RGB to HSV to allow for color separation of tissue from background and artifacts. Processing of the mask to remove small holes and objects was then performed. Following this, we iterated through the coordinates of the tissue mask to extract patches for the mutation classification dataset. Patches for use in the mutation classification task were kept if the tissue pixel fraction, as determined by the masked pixels, was at least 0.2 for a given patch. No further patch selection criteria was applied. For the *EGFR* mutation prediction task, we extracted patches at 1024 × 1024 pixels at 20 × magnification because we anticipated that the mutation classification task would require a broader view of the tumor microenvironment.

In order to perform tissue morphological profiling on the full mutation prediction patch dataset, we needed to reconcile the difference in extraction size of these patches (1024 × 1024) with the input size for the tissue morphology classification model (512 × 512 resized to 448 × 448). We did so by center-cropping each 1024 × 1024 patch to 512 × 512, resizing to 448 × 448, performing the morphology classification and then applying that classification of the center crop to the entire patch (Supplementary Fig. 2a). The tissue morphology classifications of these 1024 × 1024 patches were then used to select the appropriate patches for the two-stage models and to help analyze the MIL model’s learned attention after training.

For the training inputs of the *EGFR* mutation prediction models, we resized the raw 1024 × 1024 pixel patches to 224 × 224 pixel patches to allow each bag in the MIL formulation to include more patches, as limited by GPU memory, so that the MIL model would be allowed a more holistic view of each slide. To maintain consistency for the *EGFR* mutation classification task, we used 224 × 224 patch inputs (downsized from 1024 × 1024) for all *EGFR* mutation prediction models, including the weakly-supervised model, all two-stage models, and the MIL model (Supplementary Fig. 2b).

The *EGFR* mutation prediction task is a binary classification task where either given a single patch (in a setup where each patch has a label, such as in the mutation classification portion of the 2-stage models) or given a bag of patches (in the MIL formulation, where the bag as a whole has a label but patches individually do not) we predict from an H&E whether a gene mutation is present within a specimen. The final layer for each model is a fully-connected with a one-dimensional output and the activation function used is the sigmoid function:

$$g\left( z \right) = \frac{1}{{1 + e^{ - z} }}$$

where again

$$z = w^{T} x + b$$

The loss that we optimize for is the binary cross-entropy loss:

$$l = - (\hat{y} log\left( {g\left( z \right)} \right) + \left( {1 - \hat{y}} \right)log\left( {1 - g\left( z \right) } \right)$$

where $\hat{y}$ is the target value for the input sample. The batch loss is aggregated across each input sample within the batch by either summing or averaging the losses, and gradient descent is performed to update the model parameters. The weakly-supervised model and the two-stage models were trained for a maximum of 200 epochs with early-stopping conditioned on validation AUC. The MIL models were trained for 200 epochs with 40 patches per bag during each training pass. All models in the study were trained using the TensorFlow^33^ framework. The Adam^34^ optimizer was used with a learning rate of 1e−5.

Additionally, when running inference on the validation slides, we found that performance was notably better if batch normalization layers used batch statistics for normalization instead of using the exponentially decaying running mean and variance tracked during training. As each training step involved processing bags from at most one or two slides due to GPU memory constraints, we found that generalizability to the validation set suffered if using the standard momentum-based training statistics for batch normalization, as each batch processed would not be a sampling from the overall cohort population but rather from a very limited number of slides. If each slide during validation is processed individually, then the patch instances within each batch are drawn from the same slide and thus the instance interdependence in the batch formulation is non-arbitrary. This is analogous to vision applications extracting multiple regions-of-interest from a single image and composing those regions-of-interest into a batch to utilize batch statistics for inference^35^.

### Pathologist review of high-attention patches from high-confidence bags

To examine the attention learned by our MIL models and to better understand what features were relevant for predicting *EGFR* mutant versus wild-type specimens, expert pathologists evaluated high-attention patches for bags confidently predicted to be mutant or wild-type. For 49 validation slides, we sampled 250 patches per bag and passed each bag through the trained MIL models. The patches within each bag were then ordered by descending attention weight. The top-25 highest-attention patches for each of the 49 bags were provided to pathologists for analysis, resulting in a total of 1225 patches being reviewed.

Pathologists scored each patch for a set of numerical variables and then further reviewed each patch for categorical characteristics. The numerical variables were tumor nuclei fraction, necrosis fraction, peritumoral immune fraction, and intratumoral immune fraction. Tumor nuclei fraction was determined as the fraction of tumor nuclei relative to all nuclei present within a patch. Necrosis fraction was determined as the fraction of the patch area containing necrotic tissue. The peritumoral immune fraction was determined as the fraction of tumor edges that had noticeable immune cell response, such as lymphocytes aggregating at or within the tumor boundary. The intratumoral immune fraction was determined as the fraction of tumor tissue within a patch that had noticeable immune infiltration, such as lymphocytes dispersed throughout a tumor mass or nest.

For the review of categorical variables, pathologists examined each patch for the tumor’s predominant architectural pattern, minor architectural pattern, cytology, and any notable non-neoplastic quality. The possible predominant and minor architectural patterns were acinar, lepidic, papillary, micropapillary, mucinous, and solid. The possible cytology types were hobnail, columnar, mucinous, sarcomatoid, anaplastic, large cell, small cell, or other. Non-neoplastic qualities included fibrosis, pneumonia, inflammation, or other.

In order to evaluate overall bag characteristics relative to the model’s mutant predictions versus wild-type predictions, we generated summary statistics and overall characteristics from the pathologist review of the high-attention patches. To determine each bag’s overall numerical statistics, we calculated the mean, standard deviation, minimum, and maximum of the numerical scores provided by pathologists across the top-25 high attention patches from that bag. To determine the bag’s overall categorical characteristics, we aggregated the patch reviews across the top-attention patches by taking the mode. Thus, for each bag we had an overall summary of patch scores and categorical labels for the high-attention patches, which we could then compare based on the model’s predicted *EGFR* mutation status.

Significance between the predicted mutant and predicted wild-type slides with respect to the numerical variables was tested using the two-way T-test. False discovery rate correction was additionally applied to generate q-values from T-test *p*-values. No comparisons were significant after false discovery rate correction. Categorical comparisons were completed using the Chi-square test, at an overall bag level. Finally, association rules mining^21^ was performed by treating each overall categorical value determined for the bags as items, with predicted *EGFR* status as the consequent item-set.

### Ethics approval and consent to participate

Approval for this study, including a waiver of informed consent and a Health Insurance Portability and Accountability Act waiver of authorization, was obtained from the Western Institutional Review Board (Protocol No. 20152817).

## Supplementary Information

Supplementary Information.

## Data Availability

The datasets generated and/or analyzed during the current study are available by contacting the corresponding author. Data requestors and their institution will be required to sign a data transfer agreement. Data are not available in a public repository because they are derived from testing performed during routine patient care where rare genomic events or histology images may identify patients.
