# Supplementary material for: Predicting EGFR mutational status from pathology images using a real-world dataset
Source: Sci Rep. 2023 Mar 16;13:4404. doi: 10.1038/s41598-023-31284-6 (PMC10020556; doi:10.1038/s41598-023-31284-6)
Supplement: Supplementary file 1 — Supplementary Information. [file 41598_2023_31284_MOESM1_ESM.pdf]

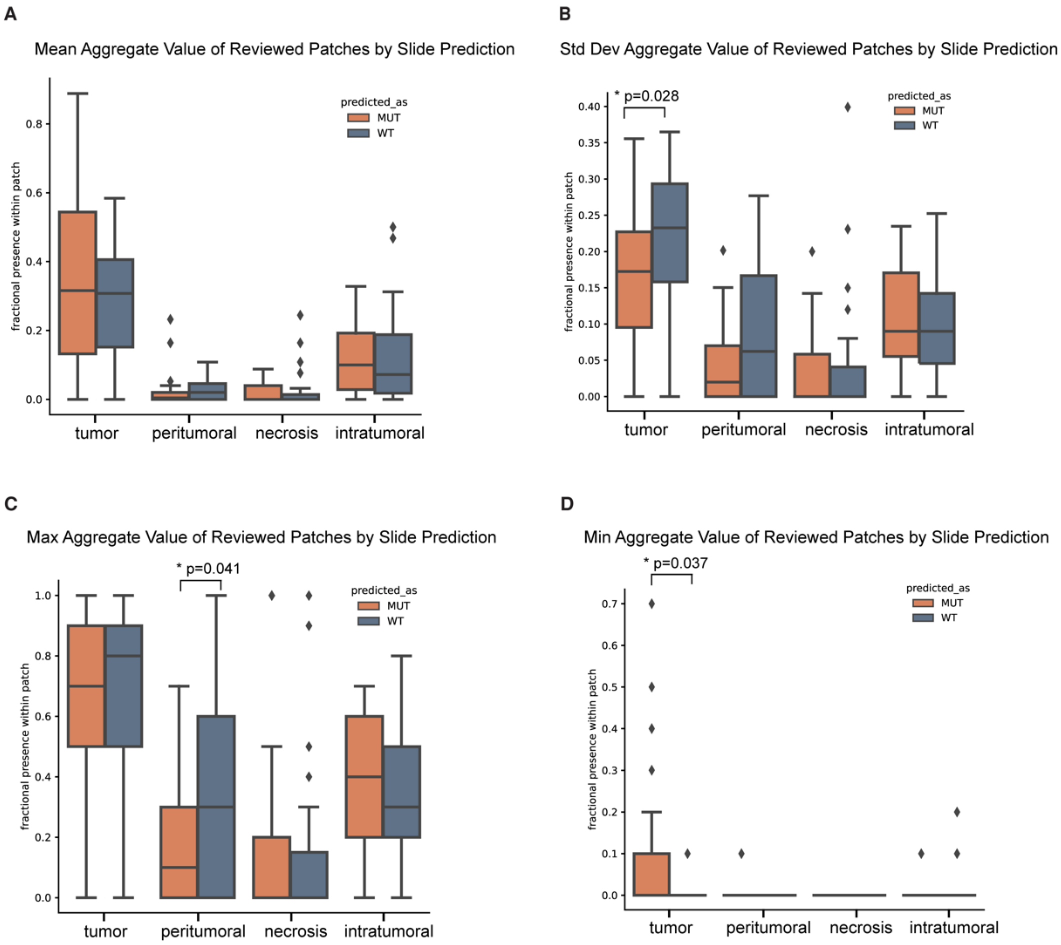


**Supplementary Figure 1. Overall bag aggregation scores of high-attention patches for numerical variables for 49 pathologist reviewed bags**.

**(a)** Mean aggregate values of the high-attention patches. **(b)** Standard deviation of the scores for the high-attention patches, with wild-type predictions having a higher standard deviation of tumor nuclei fraction than mutant calls (p=0.028). **(c)** Maximum aggregate value of the high-attention patches, with predicted wild-type bags having a significantly higher maximum peritumoral immune infiltration fraction than bags predicted to be mutant (p=0.041). **(d)** Minimum aggregate value of high-attention patches, with significantly higher minimum tumor nuclei fraction across high-attention patches for mutant predictions than wild-type predictions (p=0.037)

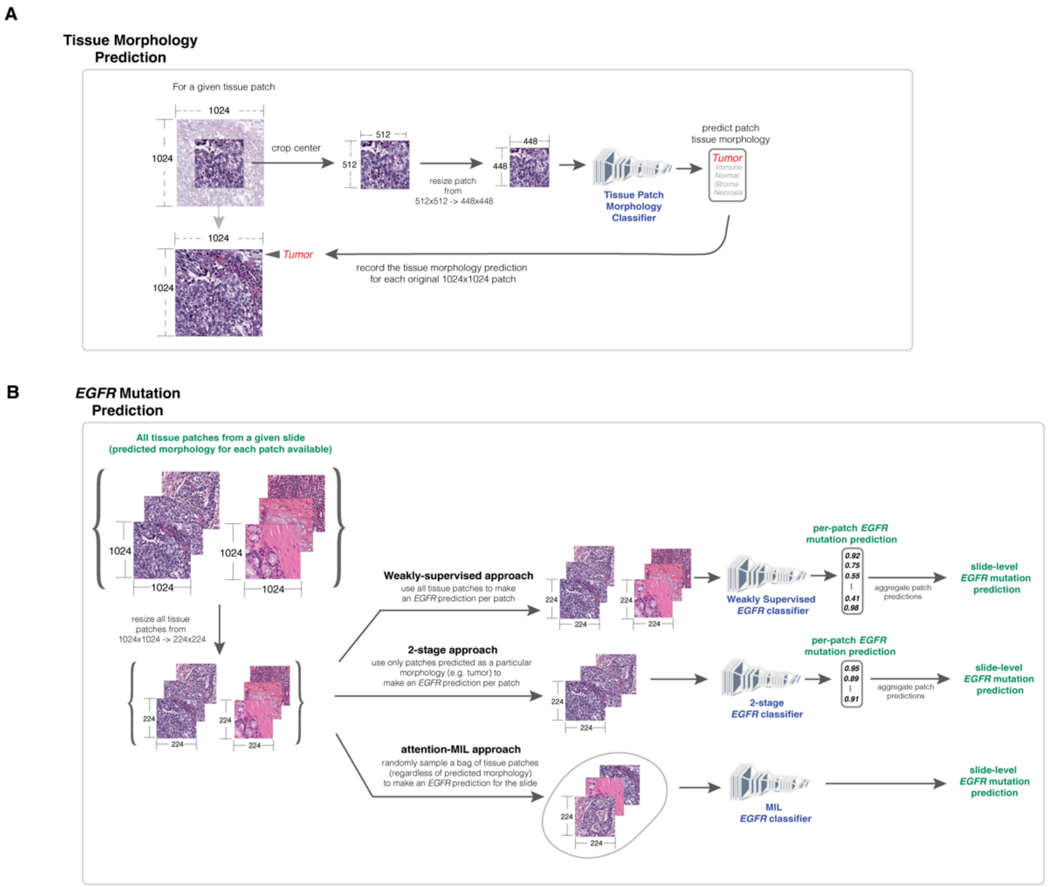


**Supplementary Figure 2. Patch processing overview of tissue morphology prediction for full patch dataset and procedure for training *EGFR* mutation prediction models**.

**(a)** Procedure for using the trained tissue morphology prediction model to classify each patch within the full dataset by taking the raw 1024x1024 patch and center cropping out a 512x512 patch, resizing that center crop patch to 448x448, making the morphology classification and then recording the classification for the raw 1024x1024 patch. **(b)** Procedure of resizing all 1024x1024 patches for which morphology predictions have been performed to 224x224 and inputting these patches into the various *EGFR* mutation prediction models, including the weakly-supervised model that uses all tissue patches from a slide, the 2-stage models that use only patches predicted to be a particular morphology type, and the MIL model that uses a random sampling of all tissue patches from the slide and for which the tissue morphology predictions may be used post-hoc to analyze learned model attention.

**Supplementary Table 1. Significance statistics of pathologist-reviewed categories relative to predicted *EGFR* mutation status for high attention patches within bags.**

| category | statistic | pvalue | rank | qvalue (practical) |
|---|---|---|---|---|
| tumor nuclei percentage_std | -2.2667706 | 0.02815071 | 1 | 0.20275584 |
| tumor nuclei percentage_min | 2.14441798 | 0.03731603 | 2 | 0.20275584 |
| peritumoral immune percentage_max | -2.1075396 | 0.04055117 | 3 | 0.20275584 |
| peritumoral immune percentage_std | -1.9727147 | 0.05455574 | 4 | 0.20458402 |
| tumor nuclei percentage_mean | 1.04516634 | 0.30140746 | 5 | 0.76205232 |
| peritumoral immune percentage_min | 0.95833333 | 0.34290476 | 6 | 0.76205232 |
| necrosis percentage_mean | -0.8391513 | 0.40572434 | 7 | 0.76205232 |
| intratumoral immune percentage_min | -0.8247776 | 0.41375449 | 8 | 0.76205232 |
| necrosis percentage_std | -0.733626 | 0.46689946 | 9 | 0.76205232 |
| tumor nuclei percentage_max | -0.6534834 | 0.5166981 | 10 | 0.76205232 |
| intratumoral immune percentage_max | 0.52140926 | 0.60458314 | 11 | 0.76205232 |
| intratumoral immune percentage_std | 0.47176191 | 0.63932689 | 12 | 0.76205232 |
| necrosis percentage_max | -0.4421601 | 0.66044535 | 13 | 0.76205232 |
| peritumoral immune percentage_mean | -0.3339032 | 0.73997008 | 14 | 0.79282508 |
| intratumoral immune percentage_mean | -0.0527459 | 0.95816281 | 15 | 0.95816281 |

**Supplementary Table 2. Association rules mining statistics using pathologist-generated categorical descriptions for high-attention patches within bags with *EGFR* predictions.**

| Antecedents | Consequents | Antecedent support | Consequent support | Support | Confidence | Lift | Leverage | Conviction |
|---|---|---|---|---|---|---|---|---|
| (fibrosis, lepidic_m, hobnail) | (EGFR+) | 0.041667 | 0.520833 | 0.041667 | 1 | 1.92 | 0.019965 | inf |
| (fibrosis, acinar_p, hobnail) | (EGFR+) | 0.041667 | 0.520833 | 0.041667 | 1 | 1.92 | 0.019965 | inf |
| (other,lepidic_p, hobnail) | (EGFR+) | 0.041667 | 0.520833 | 0.041667 | 1 | 1.92 | 0.019965 | inf |
| (columnar,acinar_p,inflammation) | (EGFR+) | 0.083333 | 0.520833 | 0.0625 | 0.75 | 1.44 | 0.019097 | 1.916667 |
| (lepidic_m,lepidic_p,hobnail) | (EGFR+) | 0.083333 | 0.520833 | 0.0625 | 0.75 | 1.44 | 0.019097 | 1.916667 |
| (columnar, papillary_p,other) | (EGFR+) | 0.0625 | 0.520833 | 0.041667 | 0.666667 | 1.28 | 0.021701 | 1.4375 |
| (lepidic_p,inflammation,hobnail) | (EGFR+) | 0.0625 | 0.520833 | 0.041667 | 0.666667 | 1.28 | 0.021701 | 1.4375 |
| **Antecedents** | **Consequents** | **Antecedent support** | **Consequent support** | **Support** | **Confidence** | **Lift** | **Leverage** | **Conviction** |
| (inflammation, hobnail, solid_m) | (EGFR-) | 0.0625 | 0.479167 | 0.0625 | 1 | 2.086957 | 0.032552 | inf |
| (inflammation, acinar_p, hobnail) | (EGFR-) | 0.0625 | 0.479167 | 0.0625 | 1 | 2.086957 | 0.032552 | inf |
| (acinar_p, hobnail, solid_m) | (EGFR-) | 0.041667 | 0.479167 | 0.041667 | 1 | 2.086957 | 0.021701 | inf |

| | | | | | | | | |
|---|---|---|---|---|---|---|---|---|
| (acinar_p, inflammation, hobnail, solid_m) | (EGFR-) | 0.041667 | 0.479167 | 0.041667 | 1 | 2.086957 | 0.021701 | inf |
| (mucinous_p, mucinous, other) | (EGFR-) | 0.083333 | 0.479167 | 0.0625 | 0.75 | 1.565217 | 0.022569 | 2.083333 |
| (acinar_p, inflammation, solid_m) | (EGFR-) | 0.0625 | 0.479167 | 0.041667 | 0.666667 | 1.391304 | 0.011719 | 1.5625 |
